# Impact of COVID-19 on the mental health of Singaporean GPs: a cross-sectional study

**DOI:** 10.3399/BJGPO.2021.0072

**Published:** 2021-07-21

**Authors:** Alvin Lum, Yen-Li Goh, Kai Sheng Wong, Junie Seah, Gina Teo, Jun Qiang Ng, Edimansyah Abdin, Margaret Mary Hendricks, Josephine Tham, Wang Nan, Daniel Fung

**Affiliations:** 1 Deputy Director, Family Physician, Institute of Mental Health, Singapore, Singapore; 2 Director, Senior Consultant, Institute of Mental Health, Singapore, Singapore; 3 Executive, Institute of Mental Health, Singapore, Singapore; 4 Senior Case Manager, Institute of Mental Health, Singapore, Singapore; 5 Senior Executive, Institute of Mental Health, Singapore, Singapore; 6 Principal Biostatistician, Institute of Mental Health, Singapore, Singapore; 7 Senior Principal Case Manager, Institute of Mental Health, Singapore, Singapore; 8 Medical Officer, Institute of Mental Health, Singapore, Singapore; 9 Chief Executive Officer, Senior Consultant, Institute of Mental Health, Singapore, Singapore

**Keywords:** COVID-19, mental health, general practitioners, primary health care, anxiety, depression, burnout, psychological, stress disorders, post-traumatic, cross-sectional studies

## Abstract

**Background:**

COVID-19 has stressed healthcare systems and workers worldwide. GPs, as first points of contact between suspected cases and the healthcare system, have assumed frontline roles in this crisis. While the prevalence of mental health problems and illnesses arising in healthcare workers (HCWs) from tertiary care settings during the COVID-19 pandemic is well-examined,^1^ the impact on GPs remains understudied.

**Aim:**

To describe the prevalence and predictors of anxiety, burnout, depression, and post-traumatic stress disorder (PTSD) among GPs during the COVID-19 pandemic.

**Design & setting:**

Survey of GPs operating in Singapore primary care clinics.

**Method:**

GPs completed a survey that comprised of four validated psychometric instruments. Open-ended questions asked about responders’ challenges and their envisaged support. Data were analysed with multiple logistic regression with demographic data as covariates; concepts of grounded theory were used to analyse the qualitative responses.

**Results:**

A total of 257 GPs participated. Fifty-five (21.4%) met the scales’ criteria for anxiety, 211 (82.1%) for burnout, 68 (26.6%) for depression, and 23 (8.9%) for PTSD. Multivariate regression analysis showed working in a public primary care setting was associated with anxiety and depression. Qualitative analyses uncovered possible stressors: changes to clinical and operational practices; increased workloads; and financial difficulties.

**Conclusion:**

Mental health issues were found to be present in Singaporean GPs during the pandemic. Prevalence of anxiety, burnout, and depression were found to be higher than those reported pre-COVID-19. The findings also provide determinants of the issues that serve as possible foci for targeted interventions.

## How this fits in

The psychological impact of COVID-19 — and its accompanying disruptions — has been documented in various populations, including HCWs. This impact on GPs, who front the fight in this pandemic, has not been examined. Singaporean GPs were recruited to answer surveys containing psychometric scales. Rates of anxiety, burnout, and depression were found to be higher than in HCWs pre-COVID-19. Risk factors for these mental health issues and possible stressors were identified. The findings provide starting points for targeted strategies to alleviate the stresses behind these problems.

## Introduction

The COVID-19 pandemic has stretched healthcare systems worldwide. Within a span of less than a year, SARS-CoV-2 — the virus responsible for the disease — infected nearly 70 million and claimed 1.5 million lives.^1,2^ Globally, clinical guidelines developed in response to this public health emergency have highlighted the importance of the GP’s role in screening and detecting for suspected cases.^3–6^ This assignment is appropriate because physicians in general practice are typically first points of contact for people with suspected COVID-19, who often exhibit influenza-like symptoms.^7^

Consequently, GPs have experienced significant changes at their workplaces including the cessation of routine services, acclimatising to new clinical practices, rationing of personal protective equipment (PPE) supplies, and being redeployed to unfamiliar workplaces. HCWs who are exposed to similar occupational disruptions, coupled with the increased risk of contracting a highly infectious disease, have been documented as having increased rates of mental health illnesses such as anxiety, depression, and PTSD, and mental health problems such as burnout.^1,8–11^

A physician workforce stricken with high rates of mental health issues has significant downstream implications. In a model produced from a review of existing research, Wallace and colleagues posited that poor physician mental health leads to poor health system outcomes,^12^ with suboptimum quality of patient care,^13^ increased medication errors,^14^ and lower productivity and efficiency.^15^ This in turn creates more workplace stressors and further deterioration of the workforce’s mental wellbeing.^12^ Detecting for these issues in physicians amid this crisis is, therefore, an immediate priority.

Current HCW mental health research is focused on those from tertiary care settings,^8,16–19^ while the mental health impact of COVID-19 on GPs remains unclear. A meta-analysis of studies conducted in hospital-based HCWs from countries affected by COVID-19 found that the majority of responders suffered mild symptoms for psychological distress associated with delivering care to diseased patients.^1^

Given the wide-ranging psychological effects of COVID-19, and that GPs have been put at the forefront of the fight in this pandemic, it is crucial to monitor rates and factors for mental health distress in these physicians to tailor timely interventions. The aim of this study, therefore, is to examine the prevalence of COVID-19-related mental health problems (burnout) and illnesses (anxiety, depression, PTSD) within a GP setting, as well as to compare the differences in sociodemographic and work environment characteristics between those with and without such issues.

## Method

### Setting

Singapore is a high-density, multiethnic city state of 5.7 million residents. Its first COVID-19 case was detected on 23 January 2020. A nationwide lockdown, colloquially termed ‘circuit breaker’, was imposed on 3 April 2020 for approximately 2 months before being gradually relaxed.^20,21^ At the time of writing (January 2021), the Singapore healthcare system has registered 58 542 cases and 29 deaths arising from the disease.^22^

Primary health care in Singapore is delivered in either publicly or privately funded settings.^23^ Public sector GPs practice at polyclinics that serve as one-stop outpatient centres with medical and allied health (diagnostic imaging and laboratory investigations, health education, and pharmacy) services. Private sector GPs typically operate in either individually owned or small-group practices. During the COVID-19 outbreak, private sector GPs who participate in the Public Health Preparedness Clinic (PHPC) scheme are provided with government support via the provision of PPE, subventions for administering national subsidy schemes related to COVID-19, grants for operating expenses incurred, and professional training to build outbreak management capacity.^24^

Local COVID-19 guidelines urge citizens who develop symptoms to seek medical attention from participating GPs (rather than at hospital emergency departments) in both public and private sectors, where fully subsidised SARS-CoV-2 polymerase chain reaction (PCR) tests are available.^6^

### Study design

The cross-sectional study took place in Singapore between April and September 2020. An earlier notification regarding the study was conveyed via email to GPs working in National University Polyclinics (NUP), a public sector network that operates six polyclinics, as well as various private primary care networks comprising of individual practice or small-group GPs. GPs had to be involved in clinical roles in either public or private primary care settings to be eligible to participate. The voluntary, anonymous, paper-based, English questionnaires were mailed to GPs who indicated interest to participate. GPs outside of these networks were also recruited through snowball sampling. A gift card was disbursed to all responders after survey completion as a token of appreciation.

Four commonly used, validated instruments were employed in the survey; relevant literature was referred to when deciding cut-offs for these scales. All instruments were validated for use,^25,26^ or previously used,^27–30^ in a Singaporean setting. Each survey also included: a section on responders’ demographic and workplace environment information; and two additional, open-ended questions asking of responders’ major concerns and envisaged support structures.

### Instruments

#### Generalised Anxiety Disorder-7 (GAD-7)

The GAD-7 is a 7-item scale that aims to measure anxiety. Items consist of anxiety-related symptoms recorded on a 4-point Likert scale. Responders were classed as being at risk of anxiety if they met the total cut-off score of 5.^31^

#### Oldenburg burnout inventory (OLBI)

The OLBI is a 16-item scale that aims to measure burnout.^32^ Items consisting of positively and negatively worded questions relating to disengagement and exhaustion are recorded on a 4-point Likert scale. Responders were classed as being at risk of burnout if they met the cut-offs of 2.25 and 2.1 for the exhaustion and disengagement subscales, respectively.^33^

#### Patient Health Questionnaire-9 (PHQ-9)

The PHQ-9 is a 9-item scale that aims to measure depression. Items consist of depression-related symptoms recorded on a 4-point Likert scale. Responders were classed as being at risk of depression if they met the total cut-off score of 5.^34^

#### Impact of Event Scale–Revised (IES-R)

The IES-R is a 22-item that aims to measure PTSD. Items measured the extent of distress in the domains of intrusion, avoidance, and hyperarousal experienced in the past week. Responders were classed as being at risk of PTSD if they met the total cut-off of 24.^35^

### Sample size

The study aimed to recruit 250 responders, which is consistent with the sample sizes in existing literature^11,29,36,37^ assessing mental health problems and illnesses among Singaporean GPs during pandemics, which have achieved between 200 and 300 responders.

### Statistics and qualitative analyses

Statistical analyses were performed using SPSS (version 25.0). The primary outcome measures were anxiety, burnout, depression, and PTSD. Explanatory variables, such as age, sex, ethnic group, marital status, type of employment, and nature of workplace environment, were included in the analyses. Multivariate logistic regression was used to assess the sociodemographic and workplace environment correlates of each dependent variable. All statistical significance was set at *P*<0.05.

In the qualitative analyses, detailed reading of the responses was done to identify signiﬁcant and recurring themes. Understanding and validating the expressed meanings of the words and phrases used in identified themes was done through research team meetings to achieve consensus. Then, verbatim quotes were assigned and arranged into themes and subthemes using the concepts of grounded theory for data analyses.^38^

## Results

### Quantitative data

The study received 257 responses (Table 1). The majority of responders were Chinese (87.2%), married (76.7%), received postgraduate (post-MBBS) medical training (76.3%), and were employed full time (84.8%). GPs from both models of practice (114 public, 137 private) and both sexes (141 male, 112 female) were well-represented. The authors identified 55 (21.4%) of the responders as having met the scales’ cut-off for anxiety, 211 (82.1%) for burnout, 68 (26.6%) for depression, and 23 (8.9%) for PTSD. Also, the mean score of each of the two subscales in the burnout instrument (OLBI) were found to be 2.72 and 2.57 for disengagement and exhaustion dimensions, respectively.

**Table 1.** Responder demographics, work environment characteristics, and measures of mental health problems and illnesses

	Overall (*n* = 257)
Age, years (mean, SD)	41.93 (12.12)	
Sex (*n*, %)		
Male	141	54.86
Female	112	43.58
No response	4	1.56
Ethnic group (*n*, %)		
Chinese	224	87.16
Malay	4	1.56
Indian	16	6.23
Eurasian	1	0.39
Other	8	3.11
No response	4	1.56
Marital status (*n*, %)		
Single	48	18.68
Married	197	76.65
Divorced or separated	6	2.33
Widowed	1	0.39
No response	5	1.95
Educational qualifications (*n*, %)		
MBBS	57	22.18
MBBS with other postgraduate qualifications	196	76.26
No response	4	1.55
Type of employment (*n*, %)		
Full time	218	84.82
Part time	17	6.61
Locum	16	6.23
Other	1	0.39
No response	5	1.95
Main workplace setting (*n*, %)		
Individual private practice	60	23.35
Shared private practice	62	24.12
PHI	124	48.25
Other	6	2.33
No response	5	1.95
PHPC scheme for clinics in private practice (*n*, %)		
PHPC	116	45.14
Non-PHPC	21	8.17
N/A (work in PHI)	114	44.36
No response	6	2.33
Clinically active years (*n*, %)		
1–3	13	5.06
4–5	27	10.51
6–10	75	29.18
11–15	30	11.67
16–20	30	11.67
≥21	77	29.96
No response	5	1.95
Number of previous outbreaks participant has been clinically involved in (*n*, %)		
0	66	25.68
1	23	8.95
2	53	20.62
3	32	12.45
4	83	32.30
Mean (SD)	2.17 (1.59)	
Scales	Meeting cut-off	
GAD-7 (*n*, %)	55	21.40
PHQ-9 (*n*, %)	68	26.56
Impact of Events-Revised (*n*, %)	23	8.94
	Mean (SD)	Meeting cut-off
Oldenburg burnout inventory		
Meeting cut-off for both disengagement and exhaustion (*n*, %)	211	82.10
Disengagement (mean score)	2.72	
Exhaustion (mean score)	2.57	

GAD-7 = Generalised Anxiety Disorder-7. PHI = public health institution. PHPC = Public Health Preparedness Clinic. PHQ-9 = Public Health Questionnaire-9

On multiple regression analyses (Tables 2–5), those who were working in the public sector polyclinics were more likely to have anxiety (odds ratio [OR] 3.42; 95% confidence interval [CI] = 1.50 to 7.77) and depression (OR 2.36; 95% CI = 1.12 to 5.00) than those working in private clinics. There were no significant relationships between other covariates and the mental health outcomes.

**Table 2.** Sociodemographic and work environment correlates of anxiety

Covariate			95% confidence interval
*P* value	Odds ratio	Lower	Upper
Age	0.18	1.04	0.98	1.11
Sex				
Male	Baseline			
Female	0.34	1.41	0.70	2.87
Ethnic group (*n*, %)				
Chinese	Baseline			
Malay	0.89	0.83	0.07	9.91
Indian	0.81	0.84	0.20	3.56
Eurasian	–	–	–	–
Other	0.52	2.07	0.22	19.21
Marital status				
Married	Baseline			
Never married	0.30	1.59	0.67	3.78
Type of employment				
Full time	Baseline			
Part time	0.46	0.58	0.14	2.47
Locum and other	0.68	0.77	0.22	2.65
Workplace setting				
Private clinic (PHPC)	Baseline			
Private clinic (Non-PHPC)	0.52	0.69	0.23	2.12
Public (polyclinic)	0.003	3.42	1.50	7.77
Number of outbreaks clinically involved in	0.40	0.88	0.65	1.19

PHPC = Public Health Preparedness Clinic.

**Table 3.** Sociodemographic and work environment correlates of burnout

Covariate			95% confidence interval
*P* value	Odds ratio	Lower	Upper
Age	0.24	1.03	0.98	1.07
Sex				
Male	Baseline			
Female	0.60	1.21	0.60	2.45
Ethnic group (*n*, %)				
Chinese	Baseline			
Malay	0.69	0.62	0.06	6.39
Indian	0.29	0.50	0.14	1.79
Eurasian	–	–	–	–
Other	–	–	–	–
Marital status				
Married	Baseline			
Never married	0.98	1.01	0.43	2.36
Type of employment				
Full time	Baseline			
Part time	0.63	1.48	0.30	7.39
Locum and other	0.91	1.08	0.28	4.14
Workplace setting				
Private clinic (PHPC)	Baseline			
Private clinic (Non-PHPC)	0.60	1.53	0.31	7.44
Public (polyclinic)	0.84	1.08	0.49	2.39
Number of outbreaks clinically involved in	0.43	1.12	0.85	1.48

PHPC = Public Health Preparedness Clinic

**Table 4.** Sociodemographic and work environment correlates of depression

Covariate			95% confidence interval
*P* value	Odds ratio	Lower	Upper
Age	0.35	1.03	0.97	1.09
Sex				
Male	Baseline			
Female	0.87	1.06	0.56	2.00
Ethnic group (*n*, %)				
Chinese	Baseline			
Malay	0.23	0.28	0.03	2.24
Indian	0.05	0.30	0.09	1.01
Eurasian	–	–	–	–
Other	0.66	0.68	0.12	3.77
Marital status				
Married	Baseline			
Never married	0.98	0.99	0.45	2.19
Type of employment				
Full time	Baseline			
Part time	0.85	1.15	0.28	4.75
Locum and other	0.56	0.71	0.22	2.24
Workplace setting				
Private clinic (PHPC)	Baseline			
Private clinic (Non-PHPC)	0.33	1.86	0.54	6.39
Public (polyclinic)	0.02	2.36	1.12	5.00
Number of outbreaks clinically involved in	0.25	1.17	0.90	1.53

PHPC = Public Health Preparedness Clinic

**Table 5.** Sociodemographic and work environment correlates of post-traumatic stress disorder

Covariate			95% confidence interval
*P* value	Odds ratio	Lower	Upper
Age	0.56	1.02	0.95	1.10
Sex				
Male	Baseline			
Female	0.25	1.82	0.65	5.09
Ethnic group (*n*, %)				
Chinese	Baseline			
Malay	–	–	–	–
Indian	0.30	0.40	0.07	2.26
Eurasian	–	–	–	–
Other	0.83	0.77	0.07	8.46
Marital status				
Married	Baseline			
Never married	0.81	1.16	0.33	4.07
Type of employment				
Full time	Baseline			
Part time	0.85	0.81	0.08	7.87
Locum and other	0.75	0.75	0.13	4.29
Workplace setting				
Private clinic (PHPC)	Baseline			
Private clinic (Non-PHPC)	0.71	0.76	0.17	3.32
Public (polyclinic)	0.11	2.61	0.81	8.43
Number of outbreaks clinically involved in	0.15	1.32	0.90	1.93

PHPC = Public Health Preparedness Clinic

### Qualitative data

Responses from open-ended questions revealed possible contributory factors to the stresses faced by GPs and support structures they found useful or hoped^39^ to receive. Three main themes of stressors emerged from the responses: (i) changed clinical and operational guidelines; (ii) increased or changed nature of workload; and (iii) financial stressors. Four types of support structures were evidenced: (i) psychosocial; (ii) clinical guidelines-related; (iii) financial; and (iv) workforce and training.

#### Stressor 1: Changed clinical and operational guidelines

Most of the responders who reported stress arising from the changed clinical and operating guidelines were private sector GPs. These changes were mandated by the country’s governing health body in response to the ongoing developments in the pandemic. Private sector GPs, who typically operate individually or in small groups, encountered difficulty in adopting these new measures and approached them apprehensively, fearing punitive enforcement:

*'Many of my GP friends feel that a lot of the stress we face in the combat of this pandemic comes from the administrative and operational aspect of the clinic. For example, the spamming of advisories from MOH* [Ministry of Health]*, the institution of SafeEntry* [a national contact tracing feature]*, the requirement to report number of patients we see, the need to apply to MTI* [Ministry of Trade and Industry] *to operate as essential service, the need to apply for number of staff, etc. All these caused me a lot more stress than clinical work. On top of it all, MOH, MTI, and NEA* [National Environmental Agency] *actually send out teams to police the clinics to ensure compliance. This is a waste of their* [workforce] *resources and creates stress for doctors. It would be better if MOH can send teams out to help us set up the various measures.* *'* (Private sector GP)

#### Stressor 2: Increased or changed nature of workload

Public sector GPs reported stress arising from the increased workloads, and physical and mental fatigue from wearing PPE for extended durations. To manage the influx of suspected cases, designated zones in polyclinics were created and staffed for isolated, prioritised care, and the higher overall workforce consumption led to temporary suspension of annual leave benefits for affected GPs:

*'*[It is] *draining when we have to wear full PPE & work in the URTI* [upper respiratory tract infection] *area. Get more staff so we can work less when we have to be in full PPE.* *'* (Public sector GP)

*'Being stretched & not able to take leave is challenging & leads to burnout.'* (Public sector GP)

*'Currently we have no way of stopping* [patient] *queues. We need a limit to our workload.* *'* (Public sector GP)

#### Stressor 3: Financial stressors

During the nationwide lockdown and the subsequent tiered re-opening phase, clinical procedures that were deemed ‘non-essential’ by local authorities, such as elective dermatological treatment or health screening services, were prohibited.^39^ Private sector GPs and GPs who worked in locum arrangements reported this temporary cessation of service offerings as a financial stressor:

*'My overriding stressor from COVID-19 is due to drop-in patient load since mid-January 2020. The actual infection & possibility of dealing with infected patients has had minimal impact.'* (Private sector GP)

*'I feel more concerned about potential loss of employment and income as a locum GP due to restrictions in number of clinics I am allowed to work at, and the recent reduction in caseload* [has] *resulted in fewer locum slots in the clinics I do locum at. Unfortunately, I do not qualify for any self-employment benefits due to my income bracket.* *'* (Private sector GP)

#### Support structure 1: Psychosocial support

GPs from both settings reported that collegial support received during or around work — via social media and messaging platforms, as well as in-person interactions — assisted their psychological wellbeing. Some responders felt a dedicated hotline for GPs seeking psychological help would be useful:

*'DOT PCN groups* [a social media group comprising GPs who participated in a nationwide primary care network initiative] *have been very valuable. Very grateful for their pointers and shared advice. Didn't feel isolated working as a solo GP practice.* *'* (Private sector GP)

*'I do think there should be a place for doctors to call in and have a dialogue to help them troubleshoot their problems instead of bottling it up and afraid to share with others for fear of being discriminated.'* (Public sector GP)

#### Support structure 2: Clinical guidelines support

GPs felt more assistance was needed to navigate the changed clinical guidelines put in place by local authorities during the pandemic. Updated information on COVID-19 from infectious disease specialist physicians would also have helped to support the GPs in their clinical duties:

*'More timely communication from policymakers about upcoming plans, and the rationale for the plans—especially when certain aspects seem counterintuitive, or when trade-offs/compromises were made for other non-medical reasons* [e.g. logistics, political, etc]*.* *'* (Private sector GP)

*'Timely updates regarding the outbreak and clarity of information* [e.g. clinical presentation of confirmed cases, transmission, progress] *from infectious disease specialists.* *'* (Public sector GP)

#### Support structure 3: Financial support

GPs from both settings felt the need to be better renumerated for their changed operating workflows during the pandemic. GPs who were in self-employment were also concerned about potential income losses and treatment costs in the event of contracting COVID-19:

*'Support financially should I be stricken/become ill for my family, staff, and hospital bills.'* (Private sector GP)

Private sector GPs espoused the need for additional subventions to offset decrease in income resulting from the mandated cessation of selected clinical services. This was despite the government’s introduction of initiatives such as the Assurance Grant, which offers monetary compensation for loss of income owing to COVID-19 illness or quarantine for eligible GPs, during the data collection:^40^

*'Financial support for private GPs because the revenue has decreased 50–70%.'* (Private sector GP)

#### Support structure 4: Workforce and training support

Public sector GPs felt the need for workforce changes, such as increased staffing to deal with suspected COVID-19 cases, to better manage their workloads. Private sector GPs wanted better training in infection control for staff in their employment:

*'Rotation between being assigned in the Red* [a designated area to manage suspected COVID-19 cases] *and Green Zone* [a designated area where patients without COVID-19 symptoms are treated] *is helpful. Having an extra day off especially after Red Zone duties to allow us to rejuvenate. More* [workforce deployed] *to the primary care departments will also help.* *'* (Public sector GP)

*'More training to manage future outbreaks. Also, train clinic staff in infection control practices.'* (Private sector GP)

## Discussion

### Summary

The study’s findings suggest that prevalences of two mental health illnesses (anxiety and depression) and one mental health problem (burnout) at the height of the COVID-19 pandemic were higher than those reported in similar demographics during earlier periods or during previous disease outbreaks. Working in public practice was identified as a significant factor for anxiety and depression. The qualitative findings evidenced that stressors for GPs were driven by changes in service delivery modes. Suggestions from GPs to better manage future outbreaks were also gathered.

### Strengths and limitations

To the authors' best knowledge, this is the first study on the impact of the COVID-19 pandemic on the mental health of GPs. Additionally, apart from determining correlated factors of the mental health issues, causative factors were also probed through qualitative inquiry. Finally, conducting this study in the months of April–September 2020, in which Singapore experienced its highest COVID-19 incidence, transmission, and mortality rates, allowed sentiments to be captured in the healthcare system during its most stressed state.

Several limitations exist. There is a paucity of research using the same (and same series of) instruments to assess for such mental health issues during pre-pandemic periods in GPs. This was owing to the numerous rating tools available, and limited the authors' ability to make statistically meaningful, empirical comparisons. It is also acknowledged that the sample size of 257 may not be representative of the sentiments of Singapore’s 8000 GPs.^41^ Sampling bias may have occurred as GPs were recruited within selected primary care networks in Singapore; furthermore, only GPs in these networks who had spare time in their demanding schedules responded. Lastly, the cross-sectional design of this study allowed only for correlations, and conclusions regarding causality cannot be made.

### Comparison with existing literature

Anxiety and depression were noted in 21.4% and 26.6% of responders, respectively. A global meta-analysis of studies investigating for these morbidities in HCWs amid the pandemic echoes the findings in the present study: pooled prevalence rates of 23.2% for anxiety and 22.8% for depression were found across 12 publications.^1^ Notably, the present study’s rates of anxiety and depression are observed to be 1.5 times and 3 times higher, respectively, when compared with rates found in a similar Singaporean study,^42^ which was conducted in the early phases of the COVID-19 pandemic (February–March 2020). This suggests that a worsened COVID-19 climate leads to increased anxiety and depression prevalence in healthcare providers, although more longitudinal data will be needed to confirm this association.

The OLBI instrument measures burnout in two dimensions: disengagement and exhaustion. Pre-pandemic studies using this instrument on similar demographics found lower severities of burnout. Mean disengagement scores assessed in HCWs worldwide during non-crisis periods ranged between 2.17 and 2.38 (versus 2.72 in the current study), while mean exhaustion scores were between 2.42 and 2.48 (versus 2.57 in the current study).^43–45^ The criteria for burnout was met by 82.1% of the responders; the following provisos are suggested when interpreting this finding. First, the prevalence should be referenced with studies using the same OLBI burnout thresholds (≥2.25 for exhaustion;≥2.10 for disengagement), because applying other thresholds found in the literature^46^ to the data may produce different findings. Second, physician burnout rates are well-documented to be high even in pre-pandemic states; for example, during medical school (44%),^47^ residency years (76%),^13^ and in full-time work (54%).^48^ Finally, the findings are similar to those of a recent, multinational study measuring HCW burnout using OLBI during COVID-19, which found an overall 67% rate of burnout and that physicians were 2.1 times more at risk than other healthcare professionals.^9^

Interestingly, rates of PTSD (8.9%) were found to be lower as compared with existing literature. Earlier studies using the IES-R to measure PTSD in HCWs during disease outbreaks estimate prevalence rates to range between 9.4% (Singaporean primary care workers during SARS) and 36.5% (Wuhan-based HCWs during the early phases of COVID-19).^8,29,49–56^ Two possible, interrelated explanations exist. First, Singaporean GPs have reported to face less social stigmatisation from friends and family owing to infection risks during COVID-19 than in previous outbreaks.^37^ This may explain the lower rates of PTSD, given that feelings of uncertainty and rejection by loved ones over contagion fears have been associated with the disorder.^37^ Second, it is postulated that GPs had sufficient PPE provided by employing institutions (for public practice) or the PHPC scheme (for private practice). This limited GPs’ viral exposure during clinical duties, which is a known risk factor found in HCW PTSD-focused literature.^30,49,53,55,57–61^ This is evidenced by the fact that 88.6% of Singaporean GPs surveyed felt they have adequate PPE.^37^ Irrespective of the exact cause, it is suggested that a tight infection control strategy can be a starting point to lower HCW PTSD rates in airborne disease pandemics.

Demographic factors were found to be significantly associated with anxiety and depression only. Unexpectedly, the findings revealed GPs working in public practice (polyclinics) were at higher risk for both morbidities, which is a comparison not previously tested for in extant research. A likely stressor unique to polyclinic GPs, which was identified from the qualitative findings, was treating a higher-than-expected volume of suspected cases. It is argued this increased proximity to the disease may have had an effect on GPs, given early evidence suggesting HCW anxiety and depression to be associated with direct diagnosis, treatment, and care of patients with COVID-19.^56,62^

While there is a high proportion of the sample that met the cut-off for burnout, the multivariate regression analyses did not find any significantly associated demographic factors. A possible explanation was the relatively small number of cases who did not meet the cut-off. This reduced the accuracy of estimation for regression coefficients and tests of significance in the logistic regression model. This effect has been documented by Peduzzi and colleagues in their work of power of predictors in low events per predictor settings.^63^

### Implications for practice

Findings discussed above highlight vulnerable segments in the GP workforce. Together with stressors and suggestions pooled in the qualitative data, two future strategies to reduce stressors behind these mental health issues are recommended for consideration. First, offering polyclinic redeployment opportunities to private sector GPs is suggested. This mobilisation serves to meet increases in patient volumes faced at these large-scale, community clinics, which should allow private GPs, whose service offerings have been trimmed by pandemic safety measures, to contribute gainfully to the national healthcare system. It should be noted that a reserve, supplementary pool of healthcare workers (Singapore Healthcare Corps) began calling for HCW volunteers in April 2020,^64^ but its effectiveness and sign-up rates from private GPs have not been published. Second, the findings also highlight the need for continued, transparent communication on pandemic-related policy changes, and assistance in managing the changed clinical guidelines from local authorities to GPs. Setting up communication channels, such as dedicated hotlines for GPs’ (clinical and non-clinical) concerns, can also be considered. Similar approaches have contributed to the effective national response in countries such as New Zealand^65,66^ and Taiwan.^67^
